# Avoiding misdiagnosis in patients with dyspnea and wheezing: a case report illustrating the clinical implications of fixation error

**DOI:** 10.1186/s12948-017-0060-9

**Published:** 2017-02-08

**Authors:** Fabiano Di Marco, Giuseppe Francesco Sferrazza Papa, Dejan Radovanovic, Pierachille Santus

**Affiliations:** 1 Respiratory Unit, ASST Santi Paolo e Carlo, Milan, Italy; 2 Dipartimento Scienze della Salute, Università degli Studi di Milano, Milan, Italy; 3 Casa di Cura del Policlinico, Dipartimento di Scienze Neuroriabilitative, Milan, Italy; 4 Lung Unit, Ospedale L. Sacco-ASST Fatebenefratelli Sacco, Via G.B. Grassi, 74, 20157 Milan, Italy

**Keywords:** Asthma, Dyspnea, Fluticasone/formoterol, Tracheal stenosis, Pulmonary function tests

## Abstract

**Background:**

Bronchial asthma is a heterogeneous respiratory condition which can be mimicked by a wide range of pathologies including upper airways stenosis. The accurate diagnosis of asthma, as with other conditions, may be influenced by fixation errors, which are common in medicine and occur when a physician concentrates on only one element of a clinical case without considering other relevant aspects. Here we report a challenging case characterized by the contemporaneous presence of a common disease, asthma, together with a rare respiratory disease, idiopathic tracheal stenosis.

**Case presentation:**

The 56-year-old female patient, a former smoker, was referred to our outpatient clinic for exertional dyspnea and persistent wheezing. There were no other respiratory or systemic symptoms over the past three months, and a psychological component was suspected. Spirometry with flow-volume evaluation and bronchoscopy were the key elements to establish the diagnoses and provide treatments. Once the diagnosis of asthma was confirmed, the combination of the anti-inflammatory corticosteroid fluticasone and the rapid-acting bronchodilator formoterol in a single inhaler effectively controlled the patient’s symptoms, confirming the favorable efficacy and safety profile which are reflected in the recommendations of the international guidelines.

**Conclusions:**

In this paper we describe the clinical investigations and interventions that eventually confirmed a diagnosis of asthma complicated by an idiopathic tracheal stenosis and led to effective treatment of the patient. Awareness of fixation error may avoid misdiagnosis in patients with respiratory disease and a complicated history at presentation.

## Background

Bronchial asthma is a heterogeneous, chronic condition affecting at least 4% of the worldwide population [1]. It is characterized by respiratory symptoms such as dyspnea, cough, and wheezing, often increasing at night and with exercise. The distinctive features of the disease are airway hyper-responsiveness to a variety of stimuli, airway inflammation, and remodeling [2]. Despite a growing number of studies, the precise pathogenesis of asthma still remains unclear. The diagnosis relies on the clinical suspicion supported by either the spirometric detection of an obstructive ventilatory defect reversible after administration of a β2-agonist, or the evidence of a variation in lung function greater than in healthy individuals [3, 4]. Symptoms and spirometric values may vary widely spontaneously or with therapy and, on average, disease control is achievable in most patients. Inhalation drugs represent the first therapeutic option and include variable doses of corticosteroids ± β2-agonist bronchodilators according to the severity of the disease [3]. A wide range of pathologies, including upper airway stenosis and vocal cord dysfunction, may mimic bronchial asthma [5, 6].

## Case presentation

A 56-year-old female patient, a former smoker with a history of 5 pack/years, was referred to our outpatient clinic for dyspnea and wheezing. Her medical history consisted of elective cholecystectomy 5 years before, a small renal cyst on sonographic follow-up, and systemic hypertension well controlled by angiotensin II receptor blockers. Her mother was affected by systemic hypertension and asthma. The patient had no history of allergies or respiratory disease. The patient complained of wheezing, present both during inspiration and expiration, and exertional dyspnea while climbing stairs, without other respiratory or systemic symptoms over the past 3 months.

**Fig. 1:**
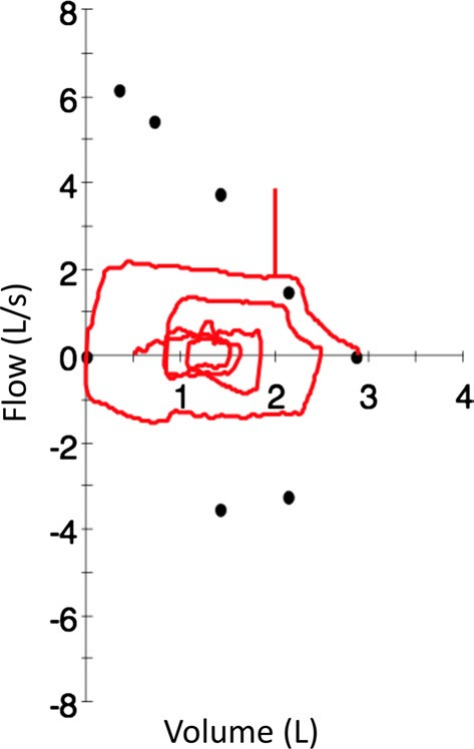
Flow-volume curve during forced maneuvers. The plateau of both the inspiratory and expiratory phases shown during forced maneuvers is suggestive of fixed central or upper airway obstruction

Due to worsening of the symptoms the patient presented to the Emergency Department, where clinical parameters were stable (oxygen saturation [SpO_2_] of 98%) and a chest X-ray showed no infiltrates, nor pleural effusion. She was discharged with a diagnosis of “anxiety”. In the following month her general practitioner, with the suspicion of asthma, empirically treated her with inhaled beclomethasone 100 mcg *b.i.d*. Due to a lack of improvement an electrocardiogram and echocardiogram were performed, which were normal, and she was subsequently sent for respiratory consultation to confirm the diagnosis of asthma. At our respiratory service the patient was eupneic at rest, with normal clinical parameters while breathing room air and well nourished (body mass index 25.0 kg/m^2^). Physical examination showed inspiratory stridor during forced inspiration. Spirometry was performed according to the ERS/ATS guidelines and showed a repeatable plateau of both the inspiratory and expiratory maneuvers, suggestive of fixed central or upper airway obstruction [4]. Forced-expiratory volume in one second (FEV_1_) was 2.03 L (83% of the predicted value), forced vital capacity (FVC) was 2.89 L (101% of the predicted value) and FEV1/FVC ratio was 0.70, 89% of the predicted value (Fig. 1).

**Fig. 2:**
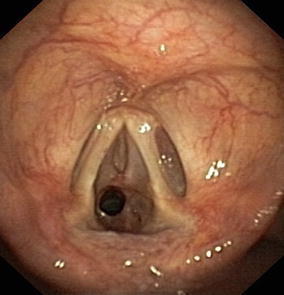
Inspective bronchoscopy revealing a subglottic tracheal stenosis

**Fig. 3:**
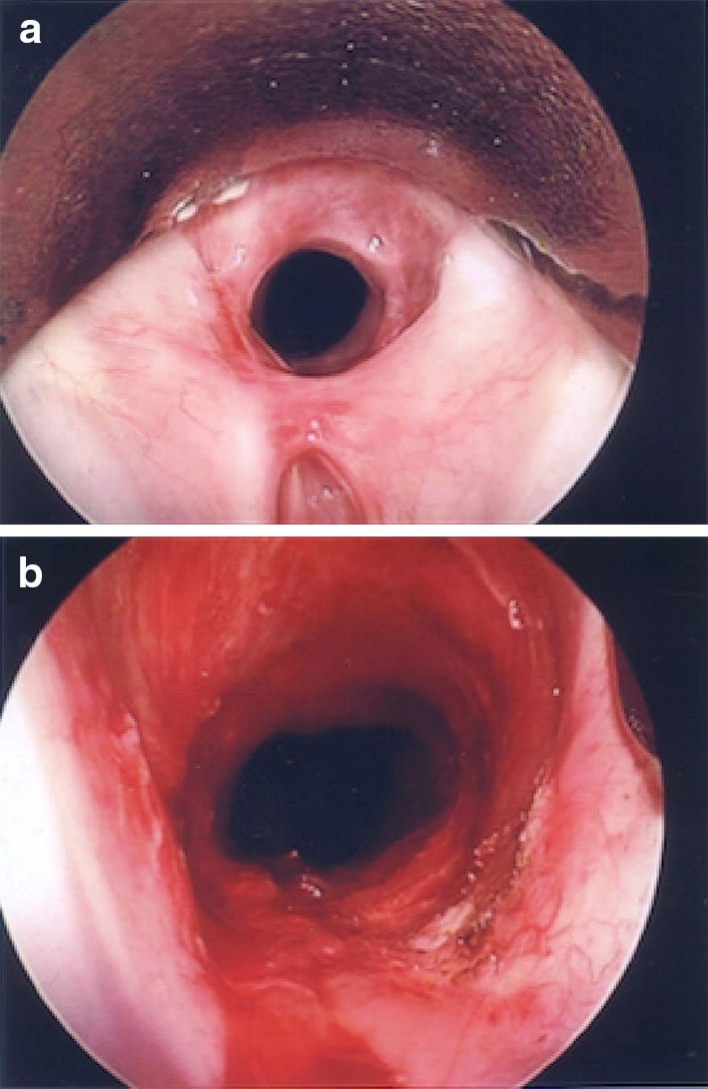
Rigid bronchoscopy showing the upper tracheal circumferential stenosis. **a** Before procedure and **b** restored patency immediately after the procedure

The patient was further evaluated with flexible bronchoscopy, which confirmed a subglottic tracheal stenosis extended for 15 mm with an internal diameter of 6 mm (Fig. 2). Autoimmunity testing was negative. The patient underwent rigid bronchoscopy with mucosal biopsies, mechanical dilatation laser-assisted (diode laser) and balloon to correct the stenosis, which reached an internal diameter of 13 mm after the procedure (Fig. 3). Biopsies showed non-specific fibro-sclerosis with no signs of vasculitis or cancer. Immediately after treatment, symptoms improved, while spirometry performed seven days after the procedure showed a normal flow volume loop with normalized obstructive indexes: FEV_1_ of 2.76 L, FVC of 3.05 L, and FEV1/FVC ratio of 0.91 (respectively, 114, 107, and 115% of the predicted values).

During follow-up at 2 and 5 months the patient presented relapses of the tracheal stenosis, due to which the endoscopic treatment was repeated with success. Seven months after the bronchoscopic treatment the patient complained of paroxysmal dyspnea, cough mainly during the night and wheezing. Bronchoscopy showed no relapse; the general practitioner suspected a psychological component due to the tracheal disease in conjunction with a recent bereavement. Since the symptoms did not stop in the following weeks, the patient was again sent to our outpatient clinic. At chest examination wheezing was detected. Baseline spirometry showed an obstructive ventilatory defect of moderate severity (FEV_1_ 1,48 L, FVC 2,68 L, and FEV1/FVC 0.55 corresponding to 61, 94 and 70% of the predicted value, respectively), with an improvement equal to 607 mL (25%) in terms of FEV_1_ after 400 mcg of inhaled albuterol via metered-dose inhaler (MDI) (FEV_1_ post bronchodilation: 2,09 L, 86% of predicted value). The suspicion of asthma was confirmed. The patient was prescribed fluticasone propionate and formoterol fumarate dihydrate at a dose of 125/5 mcg twice daily via MDI. During the subsequent 6 months of treatment she achieved normal lung function (FEV_1_ 2,76 L, FVC 3,05 L, and FEV1/FVC 0.91 corresponding to 114, 107, and 115% of the predicted value, respectively) with well controlled respiratory symptoms, and no asthma exacerbations or relapses. Accordingly, the inhalation therapy was down titrated to 50/5 mcg twice daily, with clinical stability maintained at 12 months of follow-up.

## Discussion

Respiratory symptoms are common, may overlap and are rarely specific for the underlining disease. In this case report, the patient’s history at presentation did not fit entirely with the clinical picture of bronchial asthma, due to constant symptoms with no temporal fluctuations [7]. The lack of improvement with inhaled steroids, although usually very effective for control of symptoms, does not rule out the diagnosis of asthma, since failure to improve can result from several underlining factors, including lack of compliance or incorrect use of the inhalation device. Dyspnea on exertion as the main symptom requires the exclusion primarily of chronic obstructive pulmonary disease (COPD) and chronic heart failure. Cigarette smoke is a risk factor for both diseases; however the smoking history of the patient was mild (<10 pack/years), and the presence of inspiratory stridor at clinical examination reduced the clinical plausibility of COPD. On the other hand, chronic heart failure was unlikely to be present, since arterial hypertension was well controlled with therapy, and there was no history of decompensated heart failure, or evidence of either electrocardiographic or echocardiographic alteration.

Fixation error is common in medicine, and occurs when a physician concentrates on only one aspect of a clinical case, without considering other relevant aspects [8], for example focusing on wheezing without recognizing stridor. In all chronic respiratory diseases the execution of lung function tests is of major importance to assess the presence, type and reversibility of a ventilator defect. If in some cases the test can confirm a diagnosis (e.g. in COPD), in others, such as in extra-thoracic airways obstruction, it may hint at the diagnostic pathway, e.g. suggesting a possible narrowing which should be verified by other procedures such as endoscopy. In our case, spirometry confirmed the most striking clinical information we obtained through the physical examination; stridor during forced inspiration, showing a fixed central or upper airway obstructive defect which is suspicious for the presence of laryngeal or tracheal stenosis. Notably, forced inspiratory and expiratory flow–volume curves should be repeatable and near maximal to assess these types of defects [4].

Despite the technological improvements and more skillful patient care in intensive care units, the most common benign cause of tracheal stenosis in the adult population is post-intubation laryngotracheal stenosis [9]. Our patient had a history of elective surgery (cholecystectomy); however the latter was performed 5 years before, whilst stenosis after surgery usually develops in 2 to 24 weeks after extubation [10]. Among rarer causes of tracheal lumen narrowing due to extrinsic compression are thyroid pathologies, vascular anomalies, or mediastinal lymphadenopathies, whilst intrinsic narrowing is more commonly a consequence of malignancy, tracheomalacia, granulomatosis with polyangiitis, or chronic inflammatory diseases (e.g., sarcoidosis).

Reported for the first time by Brandenburg in 1972, an idiopathic tracheal stenosis has to be suspected when an underlying etiology cannot be identified, as was the case for the patient of our report. This condition usually affects the upper trachea and occurs in the third to fifth decade of life, mostly in females [11–14]. Diagnosis of idiopathic tracheal stenosis may be difficult to achieve, and it is often made *per exclusionem* of other more common causes of dyspnea. In this case the suspicion should have been raised at physical examination, while spirometry increased the suspicion. The classic spirometric defect, a reduction in the peak expiratory flow followed by a plateau, may be seen only if the tracheal diameter is reduced to 8–10 mm. A computed tomography scan usually provides precise information regarding extent and severity of the stenosis, but ultimately the mainstay of diagnosis is represented by bronchoscopy.

The treatment of tracheal stenosis varies with the type and extent of the disease [15]. The approach is preferably surgical, entailing a single-stage laryngotracheal resection. In many cases, however, endoscopic management, laser bronchoscopy, endoscopic tracheal dilation, or stent placement is preferred [15]. In benign stenosis, a major factor for relapsing is a length of the stenosis >10 mm (15 mm in this case) [15]. Our patient was aware of the possibility of relapses, but decided for the less invasive bronchoscopic treatment.

Due to persistence and evolution of respiratory symptoms, once a relapse was excluded, the patient underwent a positive β2-stimulant reversibility test which clearly confirmed the suspicion of asthma. Although we cannot rule out that the patient was affected by asthma at the first respiratory evaluation, the clinical and functional findings were dominated by the upper airway stenosis. After the diagnosis, in consideration of the obstructive ventilatory pattern and failure of the previous treatment regimen with low dose inhaled steroids, the patient was treated with the combination of fluticasone propionate and formoterol fumarate dehydrate at medium dosage, which provides a valid therapeutic option in terms of efficacy, patient adherence to treatment and compliance to the recommendations of international guidelines, and confirmed its efficacy and safety [16–18] in this indication.

A common misdiagnosis in patients with respiratory disease is depression and anxiety. Although depression may be more common in asthmatic patients, this diagnosis and other respiratory conditions should be carefully excluded before labeling the patient as “anxious” [19–21]. However, appropriate psychiatric referral should be proposed if a psychiatric disease is suspected.

## Conclusion and teaching points

Dyspnea and wheezing are frequently associated with asthma, a disease highly prevalent in the general population. Although it is known that other diseases may mimic asthma, their recognition may not be immediate and rarer conditions, such as tracheal stenosis, may be the cause or may overlap with asthma.

Pulmonary function tests should be performed according to ERS/ATS guidelines [4, 22] and represent the first test to perform in chronic respiratory conditions to confirm a suspicion of asthma, COPD, or to suggest other diseases. Morphology of the inspiratory and expiratory flow-volume loop should be carefully inspected, since it may unveil extra-thoracic or central defects requiring specific evaluation, such as endoscopic exploration.

When assessing dyspnea, anxiety and/or depression should be diagnosed after careful exclusion of relevant respiratory and cardiovascular conditions.

## Abbreviations

COPD: chronic obstructive pulmonary disease
FEV_1_: forced-expiratory volume in one second
FVC: forced vital capacity
ICS: inhaled corticosteroid
LABA: long-acting β2-agonist
MDI: metered-dose inhaler
